# *Saccharomyces boulardii* in patients with severe acute pancreatitis: a single center, open-label randomized controlled trial

**DOI:** 10.1093/burnst/tkag006

**Published:** 2026-01-16

**Authors:** Jia-Lin He, Lei Ran, Xu Xiao, Yu Su, Hui Lin, Cheng Lu, Bo Tang, Shiming Yang

**Affiliations:** Department of Gastroenterology, Xinqiao Hospital, Army Medical University, No 183, Xinqiao Main Street, Shapingba District, Chongqing, 400037, China; Department of Gastroenterology, Xinqiao Hospital, Army Medical University, No 183, Xinqiao Main Street, Shapingba District, Chongqing, 400037, China; Department of Gastroenterology, Xinqiao Hospital, Army Medical University, No 183, Xinqiao Main Street, Shapingba District, Chongqing, 400037, China; Department of Gastroenterology, Xinqiao Hospital, Army Medical University, No 183, Xinqiao Main Street, Shapingba District, Chongqing, 400037, China; Department of Gastroenterology, Xinqiao Hospital, Army Medical University, No 183, Xinqiao Main Street, Shapingba District, Chongqing, 400037, China; Department of Gastroenterology, Xinqiao Hospital, Army Medical University, No 183, Xinqiao Main Street, Shapingba District, Chongqing, 400037, China; Department of Gastroenterology, Xinqiao Hospital, Army Medical University, No 183, Xinqiao Main Street, Shapingba District, Chongqing, 400037, China; Department of Gastroenterology, Xinqiao Hospital, Army Medical University, No 183, Xinqiao Main Street, Shapingba District, Chongqing, 400037, China

**Keywords:** *Saccharomyces boulardii;* Nosocomial infections, Respiratory and intestinal microbiota, Severe acute pancreatitis patients

## Abstract

**Background:**

Nosocomial infections in patients with severe acute pancreatitis (SAP) are frequently driven by impaired intestinal barrier function, which facilitates bacterial translocation and contributes to adverse clinical outcomes. *Saccharomyces boulardii* (*S. boulardii*) can reconstitute gut microbiota composition. We investigated whether *S. boulardii* combined with enteral nutrition (EN) affects the microbiome and nosocomial infections in SAP.

**Methods:**

This study is a single centre, open-label randomized controlled trial. We included 50 patients with SAP in a Chinese gastroenterology intensive care unit (ICU), randomized to Probiotic group (*S. boulardii* and EN) or the Control group (EN). Throat/oropharyngeal and rectal swabs were collected from patients with SAP on days 0, 1, 3, 6, 9, 12, and 15 of ICU admission. The primary endpoints were nosocomial infection and fungemia, whereas the secondary endpoints were ICU mortality, 28-day mortality, ICU stay, and length of hospital stay. All samples were subjected to full-length 16 s rRNA and internal transcribed spacer (ITS) sequencing. Multivariate analysis was performed using normalized microbial and corresponding clinical data.

**Results:**

After data processing, 213 16S rRNA and 120 ITS samples were analysed. *S. boulardii* prevented nosocomial infections (0/27 in the Probiotic group *vs* 5/23 in the Control group; *P* < 0.05). Intestinal fungi were closely associated with nosocomial infections. Bioinformatic analysis showed that *S. boulardii* prevented nosocomial infections by reducing intestinal bacterial perturbation and inhibiting the proliferation of *Enterococcus* in the intestine, and *Candida* in the respiratory tract and intestines.

**Conclusions:**

*S. boulardii* in patients with SAP may positively alter the respiratory and intestinal microbiome and decrease the incidence of nosocomial infections.

**Trial registration:**

This study was approved by the Ethics Committee of Xinqiao Hospital, Army Medical University, Chongqing China (2021-yd030–01), which was retrospectively registered at the Chinese Clinical Trial Registry (ChiCTR2200056011, Date of Registration: 30/01/2022 https://www.chictr.org.cn/showproj.html?proj=151215).

## Highlights

- *Saccharomyces boulardii* combined with enteral nutrition reduced nosocomial infections in severe acute pancreatitis.
- The probiotic intervention stabilized intestinal and respiratory microbiota composition.
- *Saccharomyces boulardii* suppressed Enterococcus in the gut and Candida in the intestine and airway.
- Gut fungal (ITS2) index strongly predicted nosocomial infection risk (AUC = 0.95).

## Background

Acute pancreatitis (AP) is one of the most common gastrointestinal causes of hospitalization, ~20% of patients progress to severe disease. These cases are often characterized by organ failure or pancreatic necrosis. Such patients typically admission to the intensive care unit (ICU) and may need therapeutic interventions to manage the complications. Nosocomial infections are among the most common complications in patients with severe acute pancreatitis (SAP) and have a significant impact on clinical outcomes [1]. These patients have often experience persistent organ failure (38%), infected pancreatic necrosis (IPN) (20%), and nosocomial infections [2–4]. Therefore, effective prevention of infection is essential.

In AP, nosocomial infections (National Healthcare Safety Network Patient Safety Component Manual, January 2025 https://www.cdc.gov/nhsn/pdfs/pscmanual/pcsmanual_current.pdf) were occurring 48 hafter admission in hospital (Hospital Day 3), such as pneumonia, line-sepsis, or bacteremia or later secondary infection of peripancreatic or pancreatic necrosis can dramatically impact clinical outcome [5]. It was often associated with gastrointestinal dysfunction and failure and are prevalent in patients [6]. Theoretically, probiotics can regulate the intestinal microbiota by competitively inhibiting the growth of pathogenic bacteria, preventing their adhesion and invasion into the intestinal epithelium, enhancing intestinal barrier function, and modulating the host immune response [7]. However, clinical research on the benefits of probiotics in patients with severe disease remains controversial. Previous studies have shown that probiotics in critically ill patients can modulate immune function, reduce the occurrence of infectious complications and ventilator-associated pneumonia (VAP), and shorten ICU hospitalization [8, 9]. However, no significant benefits were observed in morbidity or mortality rates in the ICU. Another study on SAP showed that probiotic prophylaxis did not reduce the risk of infectious complications and was associated with an increased mortality risk [10]. Furthermore, guidelines from different countries are inconsistent. The American Society for Parenteral and Enteral Nutrition (ASPEN) guidelines for nutritional support therapy in critically ill adult patients state that randomized controlled trials (RCTs) on various probiotic preparations and doses did not consistently report the outcomes included in these guidelines [11]. In contrast, the European Society for Parenteral and Enteral Nutrition guidelines on clinical nutrition in acute and chronic pancreatitis state that probiotics should not be recommended for patients with SAP [12]. Therefore, there is still no clear evidence supporting whether probiotic administration, particularly of fungal agents, affects nosocomial infections.

This study aimed to combine the widely used fungal probiotic-*Saccharomyces boulardii* (*S. boulardii*) in patients with SAP to explore their relationship with nosocomial infections and clinical prognosis. Furthermore, we aimed to elucidate the respiratory and intestinal microbial changes in nosocomial infections to provide a basis for guiding probiotic use in patients with SAP.

## Methods

### Patient inclusion and data collection

This was a single-centre, open-label RCT. Participants who met the inclusion criteria were AP was diagnosed if two of the following criteria were met: (i) lipase ≥3 times the upper limit, (ii) characteristic upper abdominal pain, and (iii) imaging features consistent with AP. According to the Revised Atlanta Classification [4], Severe AP was defined persistent organ failure (>48 h), Presence of organ failure is determined based on the Modified Marshall Scoring System. A score of 2 or more for any of 3 organ systems (respiratory, renal, or cardiovascular) defines the presence of organ failure. A total of 123 patients were diagnosed with SAP. Participants who met the inclusion criteria were further evaluated based on the following exclusion criteria: (i) nursing or pregnancy, (ii) hypersensitivity to probiotics (fungi) or other drugs, (iii) a treatment regimen including oral or intravenous antifungal drugs, (iv) an expectation of death within 48 h, or (v) an expected ICU stay of < 48 h.

This Gastroenterology ICU of university-affiliated hospital primarily admits patients with SAP from four different provinces and cities in Southwest China and provides standardized treatment throughout the disease course. It provides organ function support, endoscopic treatment of infected necrosis, and comprehensive nutritional therapy for patients with SAP. It serves as the hospital's centre for SAP management and is a member of the Chinese Acute Pancreatitis Clinical Trials Group [13–17].

### Randomization and intervention

An independent statistician, who was not involved in patient enrolment, generated a random number using SPSS 23.0 and assigned the treatments to sealed, opaque envelopes. The envelopes were sequentially coded with a continuous number.

After obtaining written informed consent from eligible patients, an independent research nurse opened the sealed envelope containing the assigned treatment within 12 h of admission to the ICU. Due to the open-label design of the trial, both patients and treating staff were aware of whether probiotics were administered. However, to reduce potential bias, investigators responsible for data collection, endpoint adjudication, and statistical analysis were blinded to group allocation.

Patients were randomly assigned to two groups: the Control group and the Probiotic group. Patients in the Control group received enteral nutrition (EN) within 24 h of ICU admission according to clinical standards: an energy intake of 12–25 kcal/kg and a protein intake of 1.2–2.0 g/kg/day (2021 Guidelines for the Provision of Nutrition Support Therapy in the Adult Critically Ill Patient by ASPEN), with the starting rate and target volume following ICU standards. Patients in the Probiotic group, in addition to conventional nutritional support, received *S. boulardii* (BIOCODEX, 0.5 g × 6 sachets/box; bedside dispensing of medications were avoided) immediately within 24 h after randomization at a dose of 0.5 g twice daily (prescribing information. Redwood City, CA: Biocodex; March 2015). Patients received probiotic therapy for 15 days during their hospital stay, even if they remained in the ICU or general ward beyond that period [18].

During clinical implementation, the study nurses were aware of the randomization details and administered *S. boulardii* to patients in the Probiotic group through jejunal feeding tubes, as per the study protocol. The jejunal tube placement was based on clinical indications rather than a study-specific requirement, and this is standard practice in our institution for patients receiving enteral therapy in the ICU.

### Outcomes

The primary endpoint was the Nosocomial infections (`Diagnostic Criteria for Hospital Infections', National Health Commission of the People's Republic of China, 2001, https://www.nhc.gov.cn/wjw/c100175/200111/59b897bdf6614e39be9ea383072a85bd.shtml; the diagnostic criteria have been translated into English and added to the supplementary materials) occurring during the index hospital admission, including respiratory, intestinal, and bloodstream infections. Respiratory infection was defined by the presence of cough with purulent sputum, moist rales, and radiologic evidence of pulmonary infiltrates on chest X-ray or CT. Throat swabs, sputum, and bronchoalveolar lavage fluid were collected for bacterial and fungal cultures. Intestinal infection was defined as fever ≥38°C, nausea, vomiting and/or abdominal pain, and diarrheal without other causes, with pathogens isolated from tissue, swab, or stool samples. Bloodstream infection (BSI) was defined as the isolation of pathogenic microorganisms from blood or catheter tip cultures.

Nosocomial infection was assessed independently by two blinded infection control experts. However, the personnel responsible for sample and data collection and the two infection control experts evaluating nosocomial infections were blinded to the group allocation. If the two specialists disagreed, the patient was not considered to have a nosocomial infection.

Secondary endpoints were ICU mortality, 28-day mortality, ICU stay, and length of hospital stay.

### Sample collection and genomic DNA extraction

Throat swabs, rectal swabs, and patient stool samples were collected separately and stored at −80°C. Genomic DNA was extracted from throat swabs, rectal swabs, and stool samples using a TIANamp DNA Kit (TIANGEN, Beijing, China) according to the manufacturer’s instructions. The concentration and purity of the extracted DNA were evaluated using a NanoDrop 2000C spectrophotometer (Thermo Fisher Scientific).

### Bioinformatics analysis

The internal transcribed spacer (ITS) sequence is located between fungal 18S, 5.8S, and 28S rRNA genes. It comprises two sub-regions, ITS1 and ITS2, with the 5.8S rRNA gene positioned between them. This region is highly valuable for molecular identification and phylogenetic analysis of fungi. In the 16S rRNA gene and ITS sequencing experiments, each sample was sequenced to a depth of 50 000 reads.

Libraries were sequenced on the Illumina NovaSeq 6000 platform (Novogene, Tianjin, China). KneadData (v0.7.4) software [19, 20] was used to process the raw sequencing data. KneadData software was run using Bowtie2 (v2.4.2) [21] to remove host genome contamination from the samples. Trimmomatic (v0.39) [22] was used to remove sequencing primers and obtain clean data. The Metaphlan3 software was used to obtain the taxonomic counts of each sample [23]. LEfSe analysis was used to identify the dominant bacteria betwcontrol groups in the bacterial and fungal community (LDA score (log10) = 4 as cutoff value).

### Statistical analysis

This study was exploratory in nature, aiming to provide a preliminary evaluation of the effect of *S. boulardii* on nosocomial infections in patients with SAP and to generate a basis for future investigations. As this was the first study conducted in a human population, parameters required for a formal sample-size calculation were unavailable. Based on the current group sizes (27 *vs* 23 patients), the incidence of nosocomial infections was 21.7% (5/23) *vs* 0% (0/27), respectively. The absolute difference between the two groups was 21.74% (95% CI, 4.39–41.90). Using PASS 21.0 with Binomial Enumeration at a two-sided α of 0.05, the calculated power was 0.86, indicating that the observed result is statistically reliable.

Clinical data were described and statistically analysed using the SPSS software (version 25.0; IBM, USA). Categorical data, such as sex, were described as frequencies and percentages (n, %), and comparisons between two groups were conducted using the chi-square or Fisher's exact test. Continuous data, such as age and ICU stay (days), were described using mean ± SD and/or median (P25–P75) depending on the distribution, with group comparisons performed using the independent samples t-test or the Mann–Whitney U test. Binary logistic regression analysis was used to identify factors associated with nosocomial infections, with *P* < 0.05 considered statistically significant. The predictive value of the ITS2 index for nosocomial infections was evaluated using receiver operating characteristic (ROC) curves and the corresponding areas under the curves (AUC).

### Institutional review board statement

This study was approved by the Ethics Committee of Xinqiao Hospital, Army Medical University, Chongqing, China, and was registered in the Chinese Clinical Trial Registry (ChiCTR2200056011, https://www.chictr.org.cn/showproj.html?proj=151215, Effect of EN supplementation with probiotics [*S. boulardii*] on new infections and intestinal microecology in ICU patients—a single-centre randomized controlled clinical study [2021-yd030–01]). This study was conducted following the principles of the 2013 Declaration of Helsinki. Written informed consent was obtained from all participants before enrolment in the study.

## Results

### Study participants and clinical characteristics

**Figure 1:**
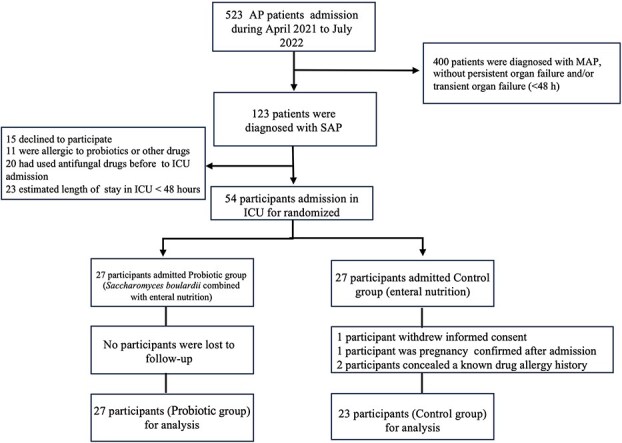
CONSORT patient flow diagram. *ICU* intensive care unit, *MAP* mild acute pancreatitis, *SAP* severe acute pancreatitis, *AP* acute pancreatitis

A total of 523 AP patients were screened between April 2021 and July 2022, of whom 123 had severe pancreatitis. 15 patients declined to participate, 11 were allergic to probiotics or other drugs, 20 had used antifungal drugs before ICU admission, and 23 had an estimated ICU stay<48 h. 54 patients admission in ICU for randomization, 27 patients in Probiotic group received *S. boulardii* combined with enteral nutrition, while 27 in Control group received EN alone (Fig. 1). In Control group, 1 patient was lost to follow-up due to withdrawal of informed consent, 1 patient was pregnancy confirmed after admission, and 2 patients’ concealment of a known drug allergy history. About 27 patients in Probiotic group and 23 patients in Control group for final analysis, and 213 16S and 120 ITS samples were included in this bioinformatics analysis.

**Figure 2:**
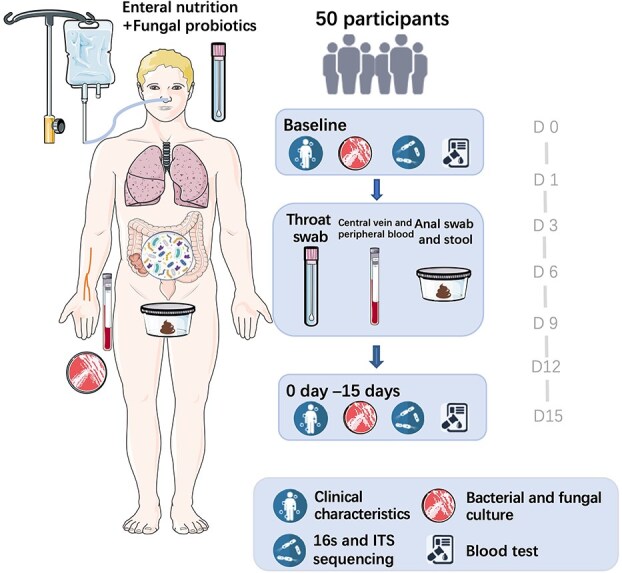
Schematic diagram of the experimental design. *ITS* internal transcribed spacer

Primary outcome data were available for all enrolled participants. Blood samples (central and peripheral veins), throat swabs, and faecal samples were collected from all eligible participants on Days 0, 1, 3, 6, 9, 12, and 15. Data regarding blood gas analysis, blood counts, biochemistry, inflammatory markers, and procalcitonin levels were collected at each time point (Fig. 2). The baseline patient characteristics showed no statistically significant differences between the Probiotic and Control groups (Table 1).

**Table 1:** Patients’ characteristics

Characteristic	Probiotic (n = 27)	Control (n = 23)	X2/F/z value	*P* value
**Age—years (mean ± SD)**	47.33 ± 12.04	52.04 ± 13.36	1.719	0.196
**Male sex (n,%)**	19 (70.37)	15 (65.22)	0.152	0.697
**APACHE-II score—median (P25-P75)**	9 (6 ~ 12)	10 (7 ~ 13)	0.713	0.476
**Marshall score (admission to ICU)**	2.00 (1.00 ~ 3.00)	4.00 (2.00 ~ 5.00)	−1.07	0.283
**Time from first symptoms to ICU admission (days)**	8 (2.50 ~ 35.00)	5 (3.00 ~ 30.00)	−0.82	0.412
**Etiology** (n,%)				
Biliary	4 (14.81)	3 (13.04)	—	1.000^#^
Alcohol	4 (14.81)	5 (21.74)	—	0.715^#^
Hypertriglyceridemia	13 (48.15)	11 (47.82)	0.001	0.982
Other	6 (22.22)	4 (17.39)	—	0.736^#^
**Source of admission to ICU** (n,%)				
Emergency department	14 (51.85)	13 (56.52)	0.109	0.857
Hospital ward	1(3.7)	2 (8.7)	—	0.588^#^
Transfer from another hospital	11 (40.74)	8 (34.78)	0.187	0.665
**Co-morbid conditions** (n,%)				
Respiratory	13 (48.15)	10 (43.48)	0.349	0.555
Cardiovascular	1 (3.7)	5 (21.74)	—	0.082
Hepatic	17 (62.96)	17 (73.91)	0.684	0.408
Renal	11 (40.74)	7 (30.43)	0.573	0.449
**Physiological support** (n,%)				
Mechanical ventilation	2 (7.41)	6 (26.09)	—	0.121
Renal replacement therapy	8 (29.63)	5 (21.74)	0.402	0.526
Plasma exchange	2 (7.41)	1 (4.35)	0	1
**CT severity index (CTSI)**	5.00 (3.00 ~ 6.50)	5.00 (3.00 ~ 7.50)	−1.14	0.256
**Patients who had infected necrotizing pancreatitis (INP** (n,%)	9 (33.33)	9 (39.13)	0.18	0.67
**Pharmacological therapy** (n,%)				
Antibiotic	15 (55.56)	15 (65.22)	0.483	0.487
Prokinetic agent	3 (11.11)	5 (21.74)	—	0.444

^#^Fisher’s Exact Test ^*^: *p* < 0.05

### Primary and secondary outcomes

The Control group had more patients with nosocomial infections (5/23 21.7% *vs* 0/27 0%, *P* = 0.016) and respiratory tract infections (4/23 17.4% *vs* 0/27 0%, *P* = 0.038) than those in the Probiotic group. One patient in the Control group had an intestinal fungal infection. All patients underwent central venous catheterization. The catheter-related BSI rates were 0% (0/27) and 13.04% (3/23) in the experimental and control groups, respectively. The infection rate in the control group was 13.04% higher than that in the probiotic group (−2.04 *vs* 32.13 95%CI) (*P* = 0.09). None of the patients had fungal BSIs (Table 2 and Supplement Table S1).

**Table 2:** Outcomes analysis

Outcome	Probiotic (n = 27)	Control (n = 23)	Median difference (95% CI)	*P* value
**Primary outcome**				
Nosocomial infection (n,%)	0 (0)	5 (21.74)	21.74 (4.39–41.90)	0.016^*^
Fungemia (n,%)	0 (0)	1 (4.35)	4.35 (−8.61–20.99)	0.46
				
**Secondary outcomes**				
ICU mortality (n,%)	0 (0)	0 (0)		—
28 days mortality (n,%)	0 (0)	0 (0)		—
ICU stay (n,%)	7.00 (6.00–9.00)	7.00 (5.00–12.00)		0.769
Length of hospital stay (n,%)	19 (14–42)	27.00 (12.00–46)		0.838
				
**Source of infection**				
Respiratory tract infection (n,%)	0 (0)	4 (17.39)	17.39 (1.16–37.14)	0.038^*^
Catheter-related blood stream infection (n,%)	0 (0)	3 (13.04)	13.04 (−2.04–32.13)	0.09
Bloodstream infection (n,%)	0 (0)	1 (4.35)	4.35 (−8.61–20.99)	0.46
Intestinal infection (n,%)	0 (0)	1 (4.35)	4.35 (−8.61–20.99)	0.46

* :*p* < 0.05

Regarding secondary outcomes, there were no statistically significant differences between the Probiotic and Control groups in ICU mortality or 28-day mortality. The median ICU length of stay was 7.0 days (IQR 6.0–9.0) in the Probiotic group and 7.0 days (IQR 5.0–12.0) in the Control group (*P* = 0.77). The total hospital length of stay was similar between two groups (median 19.0 *vs* 27.0 days, *P* = 0.84).

### Intestinal fungi are a predictor of nosocomial infections in SAP

After logistic regression analysis and adjustment for risk factors (antibiotic usage and mechanical ventilation) for nosocomial infection (Supplement Tables S2 and S3), the swab or stool ITS2 index was found to be associated with nosocomial infection (*P* <0.05, 95% CI) (Table 3). The two groups had the same diversity at baseline (Day 0, Supplement Figs. 1–3), and longitudinal analysis of nosocomial infection over time showed similar trends in each group (Supplement Figs. S4–S6). ROC curve analysis showed that the AUC of the gut fungal ITS2 index for predicting nosocomial infections was 0.950 (*P* = 0.0159) (Supplement Figure S7).

**Table 3:** Analysis of the correlation between ITS2 and nosocomial infection in severe acute pancreatitis patients

		B	SE	Wald	*P* value	OR	95% CI (low, high)
**Unadjusted**	**Constant**	1.497	1.21	1.532	0.216	4.47	
	**Swab or stool ITS2**	−1.923	0.785	5.998	**0.014** ^*^	0.146	0.031, 0.681
**Mold1**	**Constant**	−4.037	5.095	0.628	0.428	0.018	
	**Swab or stool ITS2**	−2.164	0.986	4.814	**0.028** ^*^	0.115	0.017, 0.794
**Mold2**	**Constant**	−0.611	5.554	0.012	0.912	0.543	
	**Swab or stool ITS2**	−2.179	0.987	4.873	**0.027** ^*^	0.113	0.016, 0.783
**Mold3**	**Constant**	−1.477	5.41	0.075	0.785	0.228	
	**Swab or stool ITS2**	−2.071	0.901	5.287	**0.021** ^*^	0.126	0.022, 0.737

Correction factor: Mold1: Age, sex.

Mold2: Mold 1 + Co-morbid conditions (Cardiovascular, Respiratory, Hepatic, Renal),

Mold3: Mold1+ Mold2 + APACHE II score. Antibiotic Usage,Vasoactive therapy, Mechanical ventilation, CRRT (continuous renal replacement therapy).

* :*p* < 0.05.

### Relationship of *S. boulardii* with intestinal flora balance and reduced *Enterococcus* abundance in SAP

**Figure 3:**
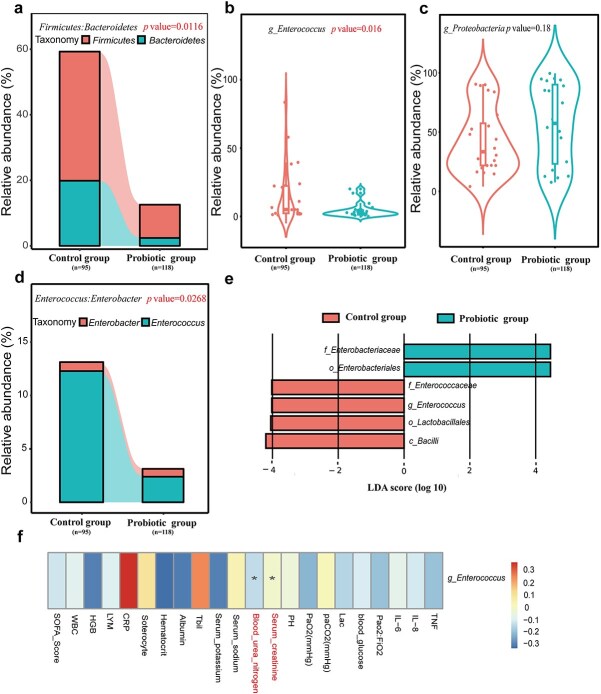
Comparison of gut microbiota between the control and probiotic groups. (**a**) Firmicutes/Bacteroidetes ratio between the control and probiotic groups (*P* < 0.05). (**b**, **c**, **d**) Relative abundances of *Enterococcus* and *Proteobacteria* (*Enterococcus/Enterobacter*) in the control and probiotic groups (*P* < 0.05). (**e**) Taxonomic representation of statistically and biologically consistent differences between the control and probiotic groups. Differences are represented by the colour of the most abundant class (red indicates the control group, green indicates the probiotic group). Histogram of LDA scores for differentially abundant genera (LDA > 4log). (**f**) Spearman correlations between *Enterococcus* and clinical outcomes as well as indicators of nosocomial infection

Differences in gut bacteria between the Probiotic and Control groups were analysed. The *Firmicutes: Bacteroidetes (F/B)* ratio decreased in the Probiotic group (*P* = 0.0116) (Fig. 3a). *Enterococcus’s* relative abundance decreased in the Probiotic group (Fig. 3b and d). The gut bacterial community at the genus level is shown as an alluvial map (Supplement Fig. S8). The Control group showed a higher abundance (LDA > 4log) of *Enterococcus* in the gut microbiota (Fig. 3e). Spearman’s correlation analysis was used to identify the relationship between gut microbiota and clinical markers, including demographic traits, laboratory tests, severity, and outcomes. *Enterococcus faecium*, a member of the *Enterococcaceae* family, was more prevalent in the Control group than in the probiotic group and positively correlated with serum creatinine but negatively correlated with blood urea nitrogen (Fig. 3f). Although the within-group comparison did not show a statistical difference (*P* = 0.15 in the Control group, *P* = 0.88 in the Probiotic group), *Enterococcus* in the gut tended to proliferate in the Control group after 48 h compared to admission to the ICU, while it showed a decreasing trend in the Probiotic group (Supplement Fig. S4).

### *S boulardii* suppresses *Candida* overgrowth in the respiratory and intestinal microbiota

**Figure 4:**
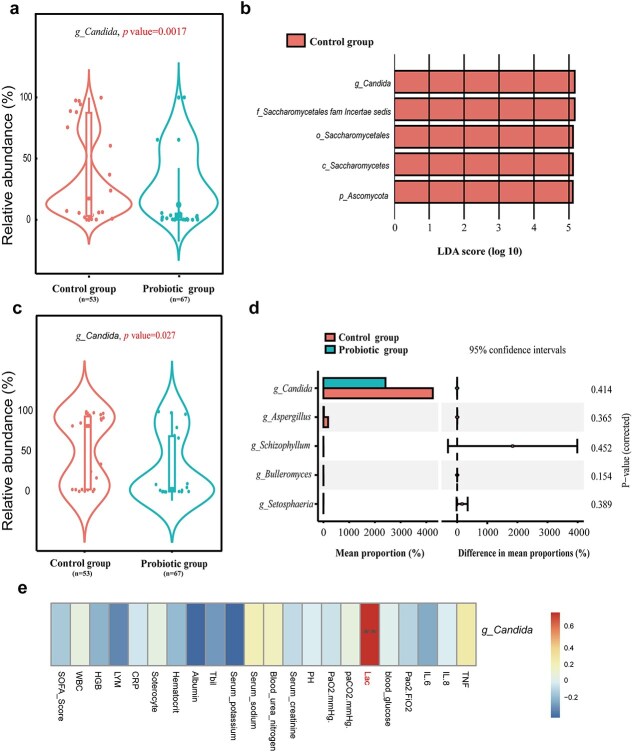
Comparison of respiratory tract and gut mycobiota between the control and probiotic groups. (**a**) Relative abundance of *Candida* in the respiratory tract between control and probiotic groups. (**b**) Taxonomic representation of statistically and biologically consistent differences between the control and probiotic groups. Differences are represented by the colour of the most abundant class (red indicates the control group). Histogram of LDA scores for differentially abundant genera (LDA > 5log). (**c**) Relative abundance of *Candida* in the gut of the control and probiotic groups. (**d**) Differentially abundant gut *Candida* species in the control and probiotic groups. Significant differences between control groups were calculated using Welch’s test, and corrected *P* values were calculated using the Bonferroni multiple test correction. Error bars indicate standard deviations of triplicate samples, and circles represent 95% confidence intervals. Red indicates the control group, and green indicates the probiotic group. (**e**) Spearman correlations between *Candida* and clinical outcomes as well as indicators of nosocomial infection

Variations in the respiratory and gut mycobiota between the Probiotic and Control groups were investigated. *Candida* proliferation decreased in the Probiotic group (Fig. 4a and c). The composition of the gut bacterial community at the genus level is shown as an alluvial map (Fig. 4d, Supplement Figure S9). The Probiotic group showed a lower abundance of Candida in the gut microbiota (LDA > 5log) (Fig. 4b). *Candida*, a member of the *Saccharomycetaceae* family, was more prevalent in the Control group than in the Probiotic group and showed a substantial positive correlation with arterial blood lactic acid (Fig. 4e). Even though the within-group comparison did not show a statistical difference (*P* = 0.32 in the Control group, and *P* = 0.33 in the Probiotic group), *Candida* in the respiratory tract tended to proliferate in the Control group after 48 h compared to admission to the ICU, whereas it showed a decreasing trend in the Probiotic group (Supplement Figure S5). Similar to the above results, the within-group comparison did not show a statistical difference (*P* = 0.72 in the Control group, *P* = 0.78 in the Probiotic group). *Candida* in the gut tended to proliferate in both the Control and Probiotic groups after 48 h compared to admission to the ICU, but its proliferation was inhibited in the Probiotic group (Supplement Figure S6).

## Discussion

In patients with SAP, the intestinal microbiota is prone to imbalances due to underlying diseases, impaired intestinal barrier function, ischemia–reperfusion injury, antibiotic use, proton pump inhibitors, and enteral or parenteral nutrition. These imbalances can lead to nosocomial infections and negatively affect the prognosis. Our study demonstrated that adding the fungal probiotic (*S. boulardii)* significantly reduced intestinal and upper respiratory tract bacteria disruptions without increasing the risk of BSI. This intervention may reduce the incidence of nosocomial infections by decreasing the proportion of intestinal *Candida*.

As reflected in alpha and beta diversity, significant differences in bacterial composition across various organs can result in bacterial translocation and an increased risk of nosocomial infections [24]. Previous studies on probiotics in patients with SAP have primarily focused on intestinal bacteria and bacterial probiotics in critically ill patients. Although intestinal fungi stabilize the intestinal mucosal barrier [7], the role of the fungal microbiota and its potential interventions in patients with SAP requires further exploration. Limited research has addressed the impact of the gut microbiota on nosocomial infections in SAP, with most studies emphasizing clinical outcomes and bacterial communities rather than fungal populations. To bridge this gap, our study utilized *S. boulardii*, the approved fungal probiotic, administered at the recommended dosage for patients with SAP to ensure safety.

We investigated the growth of *Enterobacteriaceae* and *Enterococci* in the intestines of patients with SAP and their association with nosocomial infections. Previous research has linked gut microbiota imbalances to mortality in critically ill patients, with univariate Cox regression analyses showing a strong association between *Fusobacteria* and *Enterobacteriaceae* and 180-day mortality. Multivariate analyses identified *Enterobacteriales* and *Enterobacteriaceae* as independent risk factors for 180-day mortality [25]. Other studies have linked nosocomial infection-associated bacteria, such as vancomycin-resistant *Enterococci* (VRE), to clinical outcomes. For example, reduced microbial diversity, elevated levels of pathogenic commensal bacteria and increased *Enterococcus* colonization have been identified as risk factors for carbapenem-resistant *Pseudomonas aeruginosa* (CRPA) colonization in ICU patients [26]. Additionally, a study involving 301 ICU patients found that the presence of VRE on admission was associated with a higher risk of *enterococcal* infections and a 50% increase in mortality. Even VRE-negative patients with enterococcal enrichment had a 113% increased risk of infection and death [27]. These findings highlight the close association between *Enterococci* and mortality in critically ill patients. In our study, *S. boulardii* effectively inhibited *Enterococci* proliferation in patients with SAP.

These studies also underscore the dynamic and heterogeneous nature of the gut flora in ICU patients, with distinct microbial characteristics linked to clinical outcomes. Research has increasingly focused on gut microbiota as a potential biomarker for predicting mortality and nosocomial infections in critically ill patients. Specific bacterial genera in ICU patients have been identified as predictors of sepsis outcomes, with microbiota imbalances linked to CRPA colonization and subsequent infection or death [26]. Our study found that fungal populations could effectively predict nosocomial infections in patients with SAP.

Interventions targeting gut microbiota disorders may improve the prognosis of critically ill patients. An imbalance in the intestinal flora has been linked to subsequent infection and mortality risks. Selective digestive decontamination (SDD) has been shown to reduce ICU length of stay and mortality without increasing drug resistance. However, microbiome sequencing has revealed that SDD reduces microbial diversity, increases *Enterococci* abundance, and is associated with drug-resistance genes [28]. In addition to SDD, probiotic supplementation has emerged as a promising intervention.

A retrospective analysis of 22 000 ICU patients compared outcomes between those who received *Lactobacillus rhamnosus* strain GG (LGG) and those who did not. Of the 522 patients who received LGG, 1.1% developed *Lactobacillus bacteraemia*, compared with 0.009% in the non-LGG group [29]. Another study involving 218 ICU patients compared *Lactobacillus plantarum* 299v supplementation with a placebo and found no significant differences in 60-day survival or discharge rates [30]. Similarly, a recent study showed no significant difference in the incidence of VAP between probiotic and placebo groups [31]. A meta-analysis of 65 RTCs involving 8483 critically ill patients suggested that probiotics or synbiotics might reduce VAP, hospital-acquired pneumonia, and ICU/hospital length of stay with low-certainty evidence, but they likely do not affect mortality [32]. Based on these findings, our study investigated the effects of *S. boulardii* on the respiratory and gut microbiota of patients with SAP and its potential to reduce mortality by preventing nosocomial infections.

The role of probiotics in restoring intestinal flora and improving intestinal injury in SAP has recently gained attention. Studies have shown that a mixture of six probiotic strains reduces pancreatic and intestinal damage, improves intestinal barrier function, regulates intestinal flora, and reduces bacterial translocation during AP [33, 34]. Another study reported that *Bacillus butyricus* pretreatment reduced AP severity by improving intestinal permeability and remodelling intestinal flora [35]. However, probiotics’ clinical efficacy for SAP treatment remains controversial. While some studies reported reduced SAP complications and shorter hospital stays [36], others found no significant changes in infectious complications. Notably, mesenteric ischemia-related deaths increased in one study, possibly because of high microbial loads stimulating local inflammation and reducing intestinal blood perfusion [10]. Therefore, the routine use of probiotics for SAP treatment remains controversial.

Our study focused on the effects of fungal probiotics on the intestinal microbiota of patients with SAP, emphasizing nosocomial infections and clinical outcomes. The Probiotic group experienced fewer bacterial and fungal imbalances, preserved respiratory and intestinal microbial barrier integrity, and had a reduced risk of clinical bacterial infection. Additionally, the Probiotic group showed a significantly reduced intestinal *Candida* proportion, which positively correlated with arterial blood lactate. Recent studies have highlighted the protective roles of mucosa-associated fungi such as *Candida* and *Saccharomyces* in inducing type 17 immunity and protecting against intestinal injury via IL-22-dependent mechanisms [37]. However, fungal species such as *Candida albicans* can also exacerbate conditions like ulcerative colitis under inflammatory states [38]. These findings highlight the dual role of fungi in modulating inflammation and disease severity in critically ill patients.

While our findings demonstrate significant shifts in gut microbiota composition following *S. boulardii* treatment, the precise immunological mechanisms underlying its protective effects remain to be elucidated. Prior studies suggest that *S. boulardii* can modulate immune responses, including cytokine production and enhancement of mucosal barrier function. Future studies should incorporate cytokine profiling, gut permeability assays, and histological analyses to establish a direct link between microbial modulation and host immune regulation.

This study has some limitations, including its relatively small sample size and single-centre study. Future research should employ web-based randomization to enhance concealment and involve large, multicentre RCTs to confirm these findings. Second, although the time interval between symptom onset and trial enrolment was systematically recorded, the median onset-to-enrolment duration was relatively long. Given the dynamic and time-dependent nature of severe acute pancreatitis, delayed enrolment likely corresponds to a later disease phase, during which inflammatory responses, organ dysfunction, and infectious processes may already be established. This may have attenuated the potential preventive effects of *S. boulardii*, particularly with respect to early modulation of gut barrier function and microbial translocation. Mechanical ventilation is a well-established risk factor for ventilator-associated pneumonia. Although we performed an additional subgroup analysis to examine the impact of mechanical ventilation on nosocomial infections in both groups (Supplement Table 3). Therefore, the potential confounding effect of this factor cannot be completely ruled out. The gut microbiota composition also varies based on age, sex, immune status, and ICU-acquired weakness, which may confound the correlations. Future studies should combine sequencing data with metagenomic, metabolomic, or culture-based techniques to gain a comprehensive understanding of the roles of microbial and fungal communities in SAP and nosocomial infections. Finally, fungal probiotic dosage forms for critically ill patients should be carefully considered to avoid granules in bedside preparations and minimize the risk of fungemia.

## Conclusions

Fungal probiotics in patients with SAP may influences respiratory and intestinal flora imbalances caused by other treatments and reduce the incidence of nosocomial infections. Moreover, *S. boulardii* helps preserve the intestinal fungal composition, which is closely associated with arterial blood lactate levels and may contribute to improved prognosis in patients with SAP. *S. boulardii* in patients with SAP requires further study and safety evaluation.

## Supplementary Material

supplementary-material_tkag006

## Data Availability

The datasets generated and/or analysed during this study are available at the China National Center for Bioinformation (CNCB) and are available for download via accession number CRA013183 (https://ngdc.cncb.ac.cn/gsub/submit/gsa/subCRA020895/finishedOverview). Data supporting the other findings of this study are available from the corresponding author upon reasonable request.

## Abbreviations

**AP:** acute pancreatitis

**ICU:** intensive care unit.

**SAP:** severe acute pancreatitis.

**GIDF:** gastrointestinal dysfunction and failure.

**VAP:** ventilator-associated pneumonia.

**ASPEN:** American Society for Parenteral and Enteral Nutrition.

**ESPEN:** Europe Society for Parenteral and Enteral Nutrition.

**EN:** enteral nutrition.

**GICU:** gastroenterology ICU.

**BSI:** bloodstream infection.

**NMPA:** National Medical Products Administration.

International Council for Harmonisation**:** Center for Drug Evaluation.

**IRB:** Institutional Review Board.

**CRPA:** carbapenem-resistant *P. aeruginosa*.

**VRE:** vancomycin-resistant enterococci.

**SDD:** Selective digestive decontamination.

**LGG:**  *L. rhamnosus* strain GG.

**MAF:** mucosa-associated fungi.

**UC:** ulcerative colitis.
