# Supplementary material for: Saccharomyces boulardii in patients with severe acute pancreatitis: a single center, open-label randomized controlled trial
Source: Burns Trauma. 2026 Jan 16;14:tkag006. doi: 10.1093/burnst/tkag006 (PMC12919443; doi:10.1093/burnst/tkag006)
Supplement: supplementary-material_tkag006 [file supplementary-material_tkag006.zip › Supplement Table S1.docx]

| **Supplement Table1.** Pathogens associated with different sources of infection | | | | |
| --- | --- | --- | --- | --- |
| **Infection species** | **Infection site -no** | | | |
|  | **Catheter-related blood stream infection** | **Bloodstream infection** | **Respiratory tract infection** | **Intestinal infection** |
| *Acinetobacter baumannii* | 1 |  | 2 |  |
| *Staphylococcus aureus* |  |  | 1 |  |
| *Klebsiella pneumoniae* |  |  | 1 |  |
| *Staphylococcus capitis* | 1 |  |  |  |
| *Escherichia coli* | 1 | 1 |  |  |
| *Corynebacterium striatum* |  |  | 1 |  |
| *Candida albicans* |  |  |  | 1 |
