# Supplementary material for: Saccharomyces boulardii in patients with severe acute pancreatitis: a single center, open-label randomized controlled trial
Source: Burns Trauma. 2026 Jan 16;14:tkag006. doi: 10.1093/burnst/tkag006 (PMC12919443; doi:10.1093/burnst/tkag006)
Supplement: supplementary-material_tkag006 [file supplementary-material_tkag006.zip › Supplement Table S2.docx]

| **Supplement Table2.** Antibiotic usage and nosocomial infections | | | | | |
| --- | --- | --- | --- | --- | --- |
| **Antibiotic usage** | **Nosocomial infections** | **Total (n=50)** | **Probiotic (n=27)** | **Control (n=23)** | ***P* value*** |
| Without antibiotic usage (n, %) | No | 19 (95.00) | 12 (100.00) | 7 (87.50) | 0.400 |
|  | Yes | 1 (5.00) | 0 (0) | 1 (12.50) |  |
|  | Total | 20 (40.00) | 12 (44.44) | 8 (34.78) |  |
| With antibiotic usage (n, %) | No | 26 (86.67) | 15 (100.00) | 11 (73.33) | 0.050 |
|  | Yes | 4 (13.33) | 0 (0) | 4 (26.67) |  |
|  | Total | 30 (60.00) | 15 (55.56) | 15 (65.22) |  |
| Total (n, %) | No | 45 (90.00) | 27 (100.00) | 18 (78.26) | 0.016* |
|  | Yes | 5 (10.00) | 0 (0) | 5 (21.74) |  |
| *: *p*<0.05 |  |  |  |  |  |
