# Supplementary material for: Saccharomyces boulardii in patients with severe acute pancreatitis: a single center, open-label randomized controlled trial
Source: Burns Trauma. 2026 Jan 16;14:tkag006. doi: 10.1093/burnst/tkag006 (PMC12919443; doi:10.1093/burnst/tkag006)
Supplement: supplementary-material_tkag006 [file supplementary-material_tkag006.zip › Supplement Table S3.docx]

| **Supplement Table3.** Mechanical ventilation and nosocomial infections | | | | | |
| --- | --- | --- | --- | --- | --- |
| **Mechanical ventilation** | **Nosocomial infections** | **Total (n=50)** | **Probiotic (n=27)** | **Control (n=23)** | ***P* value*** |
| Without mechanical ventilation (n, %) | No | 41 (97.62) | 25 (100.00) | 16 (94.12) | 0.406 |
|  | Yes | 1 (2.38) | 0 (0) | 1 (5.88) |  |
| Total |  | 42 (84.00) | 25 (92.59) | 17 (73.91) |  |
| With mechanical ventilation (n, %) | No | 4 (50.00) | 2 (100.00) | 2 (33.33) | 0.429 |
|  | Yes | 4 (50.00) | 0 (0) | 4 (66.67) |  |
| Total |  | 8 (16.00) | 2 (7.41) | 6 (26.09) |  |
| Total (n, %) | No | 45 (90.00) | 27 (100.00) | 18 (78.26) | 0.016* |
|  | Yes | 5 (10.00) | 0 (0) | 5 (21.74) |  |
| *: *p*<0.05 |  |  |  |  |  |
