# Supplementary material for: Saccharomyces boulardii in patients with severe acute pancreatitis: a single center, open-label randomized controlled trial
Source: Burns Trauma. 2026 Jan 16;14:tkag006. doi: 10.1093/burnst/tkag006 (PMC12919443; doi:10.1093/burnst/tkag006)
Supplement: supplementary-material_tkag006 [file supplementary-material_tkag006.zip › supplements_20260108_tkag006.pdf]

**Diagnostic Criteria for Hospital Infections**
**(National Health Commission of the People's Republic of China, 2001)**

Hospital infections (Nosocomial Infection, Hospital Infection, or Hospital-acquired Infection) refer to infections acquired by inpatients during their hospital stay. This includes infections that occur during hospitalization and those acquired in the hospital but manifest after discharge. It excludes infections that began before admission or were present at the time of admission. For infections without a definite incubation period, infections occurring 48 hours after admission are considered hospital infections.

**Respiratory infections** are defined as follows:
1. Upper Respiratory Tract Infections (URTI):
- Clinical Diagnosis: Fever (⩾38.0° C for more than 2 days) with acute inflammatory signs in the upper respiratory tract, including the nasopharynx, paranasal sinuses, and tonsils.
- Etiological Diagnosis: Based on clinical diagnosis, significant pathogenic microorganisms can be identified through smear or culture of secretions.
- Note: Common cold and acute inflammation of the upper respiratory tract caused by non-infectious factors (e.g., allergies) must be excluded.

2. Lower Respiratory Tract Infections (LRTI):
- Clinical Diagnosis: Diagnosis can be made if either of the following criteria is met:the patient presents with cough and thick sputum, with moist rales in the lungs, and any of the following:
  i. Fever.
  ii. Increased total white blood cell count and/or neutrophil proportion.
  iii. X-ray shows inflammatory infiltrative lesions in the lungs.

**Blood infections** are as follows:
1.Catheters-Related Blood Streaming Infections:
Clinical Diagnosis: The diagnosis can be made if any one of the following three criteria is met:
  i. Pus discharge at the venipuncture site or diffuse erythema (indicative of cellulitis).
  ii. Painful diffuse erythema along the subcutaneous path of the catheter, excluding physical or chemical causes.
  iii. Fever >38°C after vascular intervention, with local tenderness and no other explanatory cause.

Etiological Diagnosis: Isolation of significant pathogenic microorganisms from the catheter tip culture and/or blood culture.
Notes:
i. For catheter tip culture, the catheter tip should be cultured by rolling the distal 5 cm on a blood agar plate. A positive result is indicated by ⩾15 CFU per plate.
ii. Quantitative blood culture from the puncture site showing ⩾100 CFU/mL, or a bacterial count 4-10 times higher than that from a simultaneous contralateral blood culture; or the same bacteria isolated from simultaneous contralateral blood culture.

2. Sepsis:
Clinical Diagnosis: Diagnosis can be made if the patient has a fever >38°C or hypothermia <36°C, possibly with chills, along with any of the following conditions:

i. Presence of an entry portal or metastatic focus of infection.
ii. Systemic toxic symptoms without a clear infection focus.
iii. Presence of rash or petechiae, hepatosplenomegaly, increased neutrophils with left shift, and no other explanatory cause.
iv. Systolic blood pressure below 12 kPa (90 mmHg) or a drop in systolic pressure exceeding 5.3 kPa (40 mmHg) from baseline.

Etiological Diagnosis: Based on clinical diagnosis, the presence of one of the following two criteria confirms the diagnosis:

i. Isolation of pathogenic microorganisms from blood culture.
ii. Detection of pathogenic antigens in the blood.

**Intestinal infections** are as follows:

Clinical Diagnosis: The diagnosis can be made if the patient exhibits fever (≥38° C), nausea, vomiting, and/or abdominal pain, diarrhea, with no other explanatory cause.

Etiological Diagnosis: Based on the clinical diagnosis, the presence of any one of the following three criteria confirms the diagnosis:

i. Pathogens isolated from tissue specimens obtained via surgical or endoscopic procedures, or from surgical drainage fluid cultures.
ii. Presence of pathogens or multinucleated giant cells seen in Gram-stained or potassium hydroxide preparations of the aforementioned specimens.
iii. Histopathological evidence of infection in specimens obtained through surgery or endoscopy.

**16S rRNA and ITS gene amplification by PCR**

The primers specific to the V3-V4 region of the 16S rRNA gene were 341F (CCTAYGGGR BGCASCAG) and 806 R (GGACTACNNGGGTATCTAAT).

The primers specific to the ITS2 region of ITS gene were ITS3-2024F(GCATCGATGAAGA ACGCAGC) and ITS4-2409R(TCCTCCGCTTATTGATATGC).

All PCR reactions were carried out with 15μL of Phusion® High-Fidelity PCR Master Mix (New England Biolabs); 0.2 μM of forward and reverse primers, and about 10 ng template DNA. Thermal cycling consisted of initial denaturation at 98°C for 1 min, followed by 30 cycles of denaturation at 98°C for 10 s, annealing at 50°C for 30 s, and elongation at 72° C for 30 s. Finally 72℃ for 5 min.

**Collection Protocol for Respiratory and Intestinal Samples for 16S rRNA Sequencing**

| Sampling time points (ICU stay) | | Number of samples collected | | | | | | Number of samples submitted for testing | | | | | | Number of samples included in the analysis | | | | | |
|---|---|---|---|---|---|---|---|---|---|---|---|---|---|---|---|---|---|---|---|
| | | Respiratory tract | | | Intestinal | | | Respiratory tract | | | Intestinal | | | Respiratory tract | | | Intestinal | | |
| | | Control (N=23) | Probiotic (N=27) | Total | Control (N=23) | Probiotic (N=27) | Total | Control (N=23) | Probiotic (N=27) | Total | Control (N=23) | Probiotic (N=27) | Total | Control (N=23) | Probiotic (N=27) | Total | Control (N=23) | Probiotic (N=27) | Total |
| Baseline | Baseline Day 0 | 23 | 27 | 50 | 23 | 27 | 50 | 23 | 27 | 50 | 23 | 27 | 50 | 23 | 27 | 50 | 23 | 27 | 50 |
| | Baseline Day 1 | | | | | | | | | | | | | | | | | | |
| Nosocomial infection occurred time | Day 3 | 23 | 27 | 50 | 23 | 27 | 50 | 23 | 27 | 50 | 23 | 27 | 50 | 23 | 27 | 50 | 23 | 27 | 50 |
| | Day 6 | 20 | 24 | 44 | 20 | 24 | 44 | 20 | 24 | 44 | 20 | 24 | 44 | 20 | 24 | 44 | 20 | 24 | 44 |
| | Day 9 | 17 | 20 | 37 | 17 | 20 | 37 | 17 | 20 | 37 | 17 | 20 | 37 | 16 | 19 | 35 | 16 | 19 | 35 |
| | Day 12 | 10 | 15 | 25 | 10 | 15 | 25 | 10 | 15 | 25 | 10 | 15 | 25 | 10 | 15 | 25 | 10 | 15 | 25 |
| | Day 15 | 3 | 6 | 9 | 3 | 6 | 9 | 3 | 6 | 9 | 3 | 6 | 9 | 3 | 6 | 9 | 3 | 6 | 9 |
| | Total | 96 | 119 | 215 | 96 | 119 | 215 | 96 | 119 | 215 | 96 | 119 | 215 | 95 | 118 | 213 | 95 | 118 | 213 |

**Collection Protocol for Respiratory and Intestinal Samples for ITS Sequencing**

| Sampling time points (ICU stay) | | Number of samples collected | | | | | | Number of samples submitted for testing | | | | | | Number of samples included in the analysis | | | | | |
|---|---|---|---|---|---|---|---|---|---|---|---|---|---|---|---|---|---|---|---|
| | | Respiratory tract | | | Intestinal | | | Respiratory tract | | | Intestinal | | | Respiratory tract | | | Intestinal | | |
| | | Control (N=23) | Probiotic (N=27) | Total | Control (N=23) | Probiotic (N=27) | Total | Control (N=23) | Probiotic (N=27) | Total | Control (N=23) | Probiotic (N=27) | Total | Control (N=23) | Probiotic (N=27) | Total | Control (N=23) | Probiotic (N=27) | Total |
| Baseline | Baseline Day 0 | 23 | 27 | 50 | 23 | 27 | 50 | 23 | 27 | 50 | 23 | 27 | 50 | 19 | 21 | 40 | 18 | 21 | 39 |
| | Baseline Day 1 | | | | | | | | | | | | | | | | | | |
| Nosocomial infection occurred time | Day 3 | 23 | 27 | 50 | 23 | 27 | 50 | 23 | 27 | 50 | 23 | 27 | 50 | 15 | 18 | 33 | 15 | 18 | 33 |
| | Day 6 | 20 | 24 | 44 | 20 | 24 | 44 | 20 | 24 | 44 | 20 | 24 | 44 | 10 | 12 | 22 | 10 | 12 | 22 |
| | Day 9 | 17 | 20 | 37 | 17 | 20 | 37 | 17 | 20 | 37 | 17 | 20 | 37 | 6 | 9 | 15 | 6 | 9 | 15 |
| | Day 12 | 10 | 15 | 25 | 10 | 15 | 25 | 10 | 15 | 25 | 10 | 15 | 25 | 3 | 5 | 8 | 3 | 5 | 8 |
| | Day 15 | 3 | 6 | 9 | 3 | 6 | 9 | 3 | 6 | 9 | 3 | 6 | 9 | 1 | 2 | 3 | 1 | 2 | 3 |
| | Total | 96 | 119 | 215 | 96 | 119 | 215 | 96 | 119 | 215 | 96 | 119 | 215 | 54 | 67 | 121 | 53 | 67 | 120 |

## Supplement Table1 Pathogens associated with different sources of infection

| Infection species | Infection site -no | | | |
| --- | --- | --- | --- | --- |
| | Catheter-related blood stream infection | Bloodstream infection | Respiratory tract infection | Intestinal infection |
| Acinetobacter baumannii | 1 | | 2 | |
| Staphylococcus aureus | | | 1 | |
| Klebsiella pneumoniae | | | 1 | |
| Staphylococcus capitis | 1 | | | |
| Escherichia coli | 1 | 1 | | |
| Corynebacterium striatum | | | 1 | |
| Candida albicans | | | | 1 |

**Supplement Table2 Antibiotic usage and nosocomial infections**

| Antibiotic usage | Nosocomial infections | Total (N=50) | Probiotic (N=27) | Control (N=23) | P-value* |
|---|---|---|---|---|---|
| Without antibiotic usage (n,%) | No | 19 (95.00) | 12 (100.00) | 7 (87.50) | 0.400 |
| | Yes | 1 (5.00) | 0 (0) | 1 (12.50) | |
| | Total | 20 (40.00) | 12 (44.44) | 8 (34.78) | |
| With antibiotic usage (n,%) | No | 26 (86.67) | 15 (100.00) | 11 (73.33) | 0.050 |
| | Yes | 4 (13.33) | 0 (0) | 4 (26.67) | |
| | Total | 30 (60.00) | 15 (55.56) | 15 (65.22) | |
| Total (n,%) | No | 45 (90.00) | 27 (100.00) | 18 (78.26) | 0.016* |
| | Yes | 5 (10.00) | 0 (0) | 5 (21.74) | |

*:$p<0.05$

**Supplement Table3 Mechanical ventilation and nosocomial infections**

| Mechanical ventilation | Nosocomial infections | Total (N=50) | Probiotic (N=27) | Control (N=23) | P-value* |
|---|---|---|---|---|---|
| Without mechanical ventilation (n,%) | No | 41 (97.62) | 25 (100.00) | 16 (94.12) | 0.406 |
| | Yes | 1 (2.38) | 0 (0) | 1 (5.88) | |
| Total | | 42 (84.00) | 25 (92.59) | 17 (73.91) | |
| With mechanical ventilation (n,%) | No | 4 (50.00) | 2 (100.00) | 2 (33.33) | 0.429 |
| | Yes | 4 (50.00) | 0 (0) | 4 (66.67) | |
| Total | | 8 (16.00) | 2 (7.41) | 6 (26.09) | |
| Total (n,%) | No | 45 (90.00) | 27 (100.00) | 18 (78.26) | 0.016* |
| | Yes | 5 (10.00) | 0 (0) | 5 (21.74) | |

*:$p<0.05$

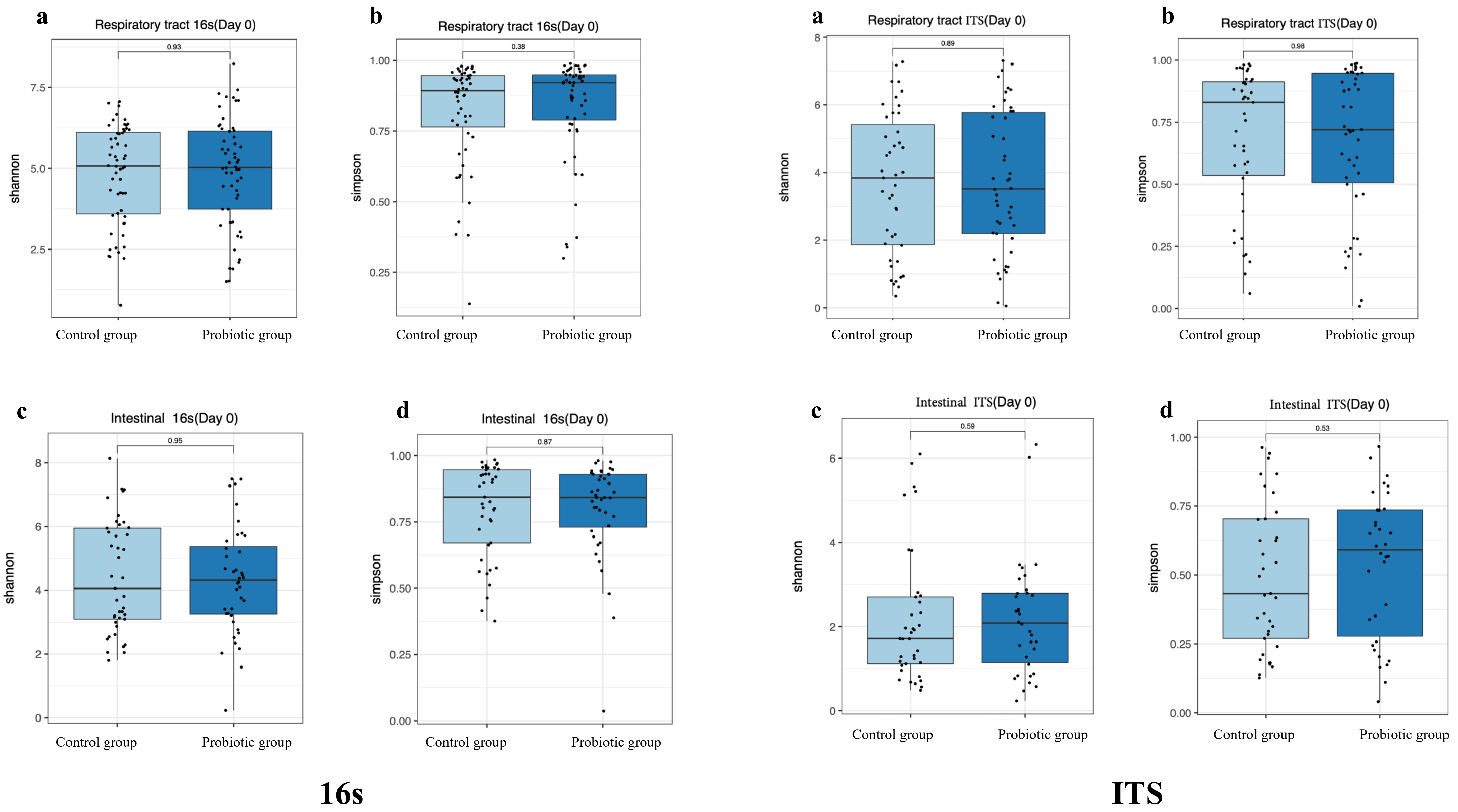


**Supplement Fig 1 Baseline α-Diversity of 16S and ITS in Respiratory and Intestinal Samples**

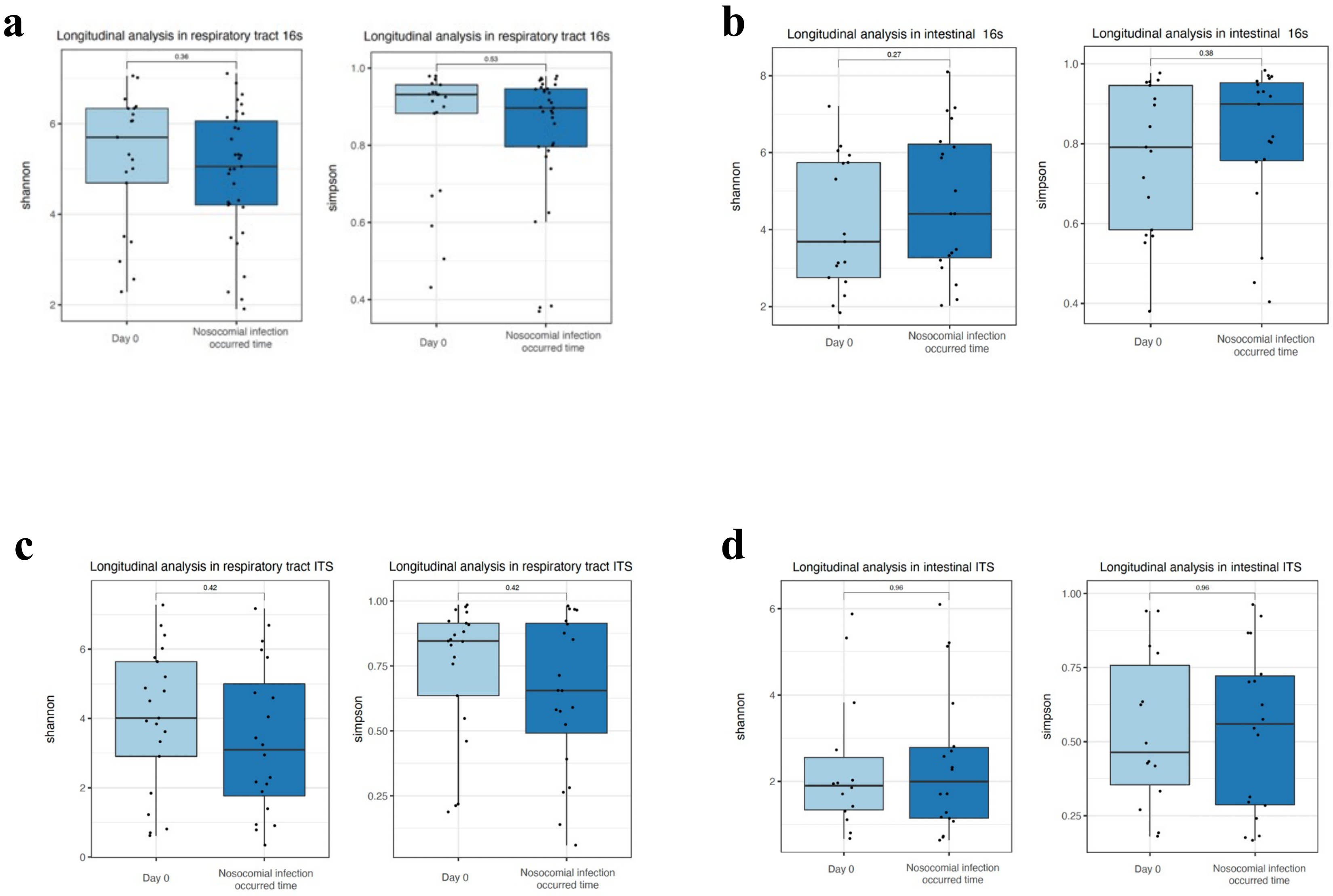


**Supplement Fig 2 . α-Diversity of 16S and ITS in Respiratory and Intestinal Samples longitudinal analysis in Control group**

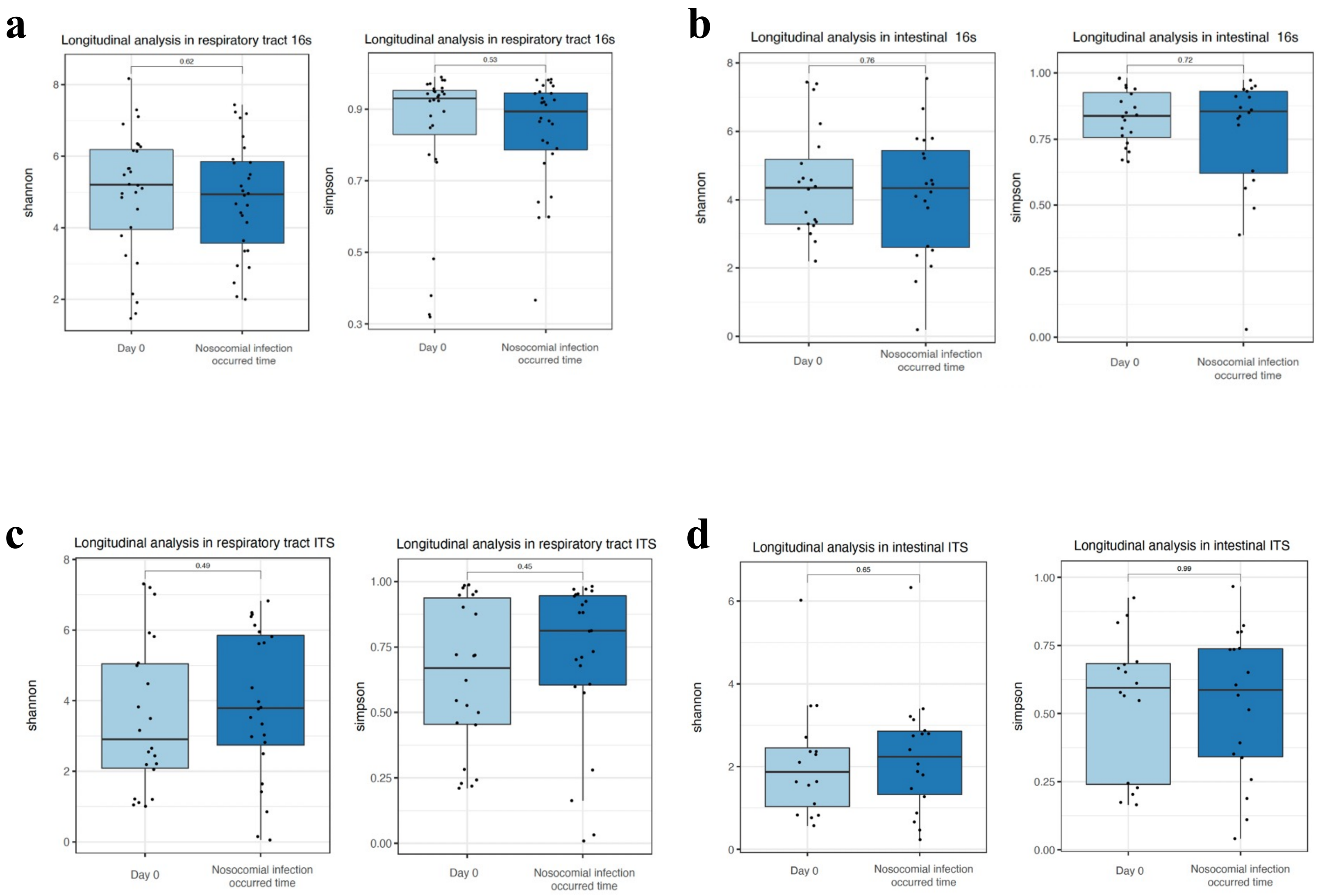


**Supplement Fig 3. α-Diversity of 16S and ITS in Respiratory and Intestinal Samples longitudinal analysis in Probiotic group**

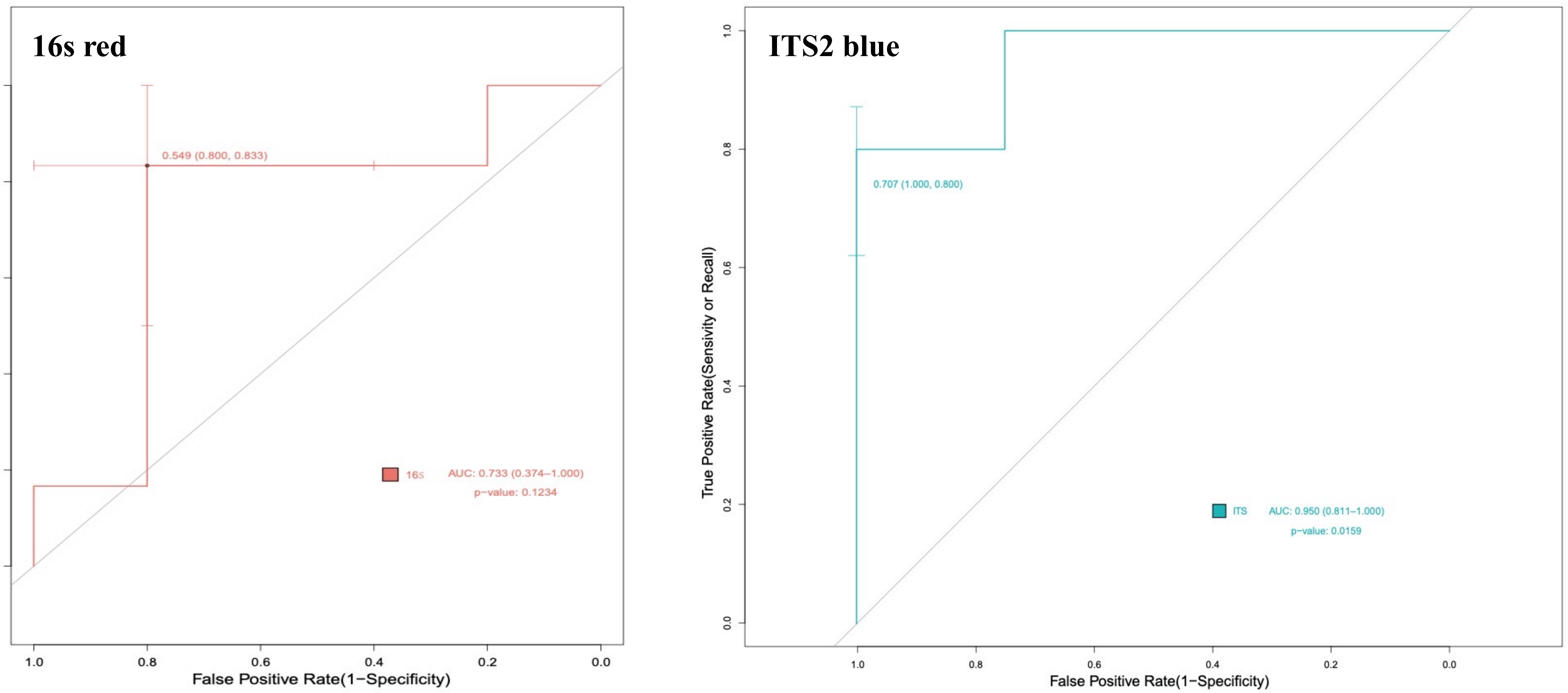


**Supplement Fig 4. ROC curve analysis of the gut bacteriobiota（16s red） and mycobiota（ITS2 blue） diagnosis index for predicting nosocomial infection among severe acute pancreatitis patients.**

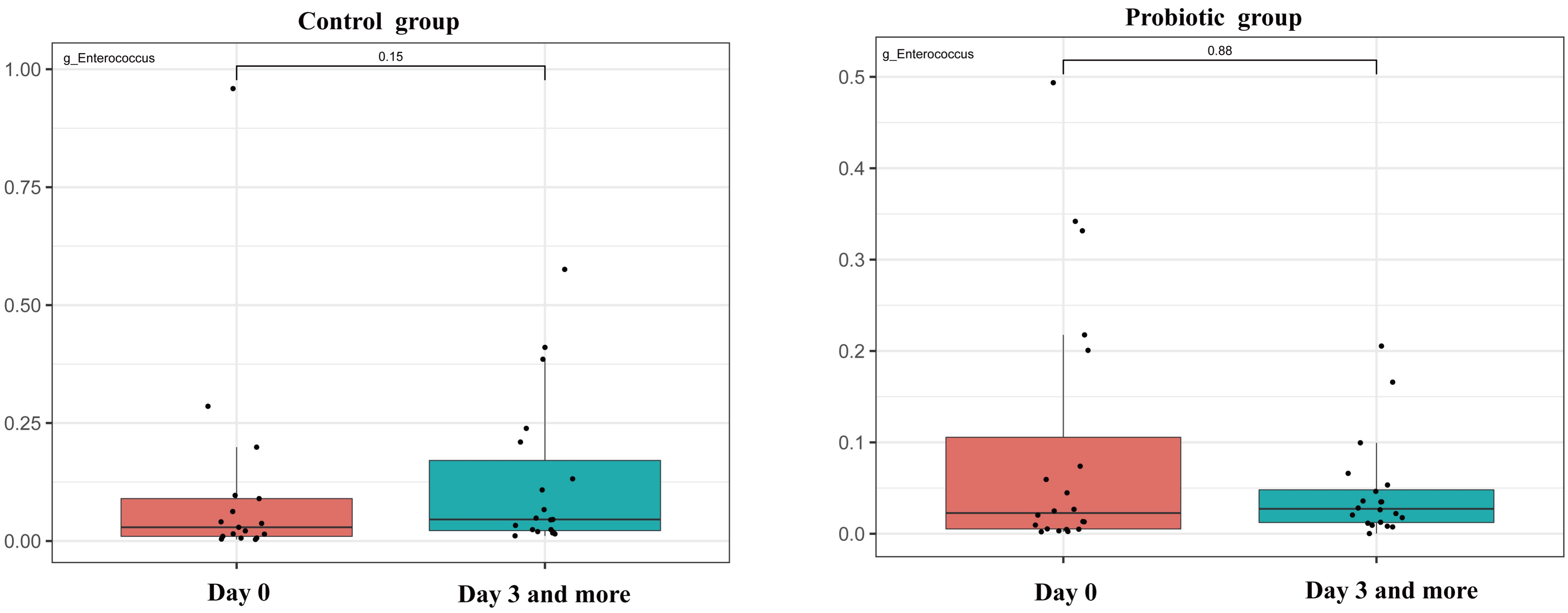


**Supplement Fig 5 The proliferation of Enterococcus in gut from administration into ICU and more than 48 hours between Control group and Probiotic group.**

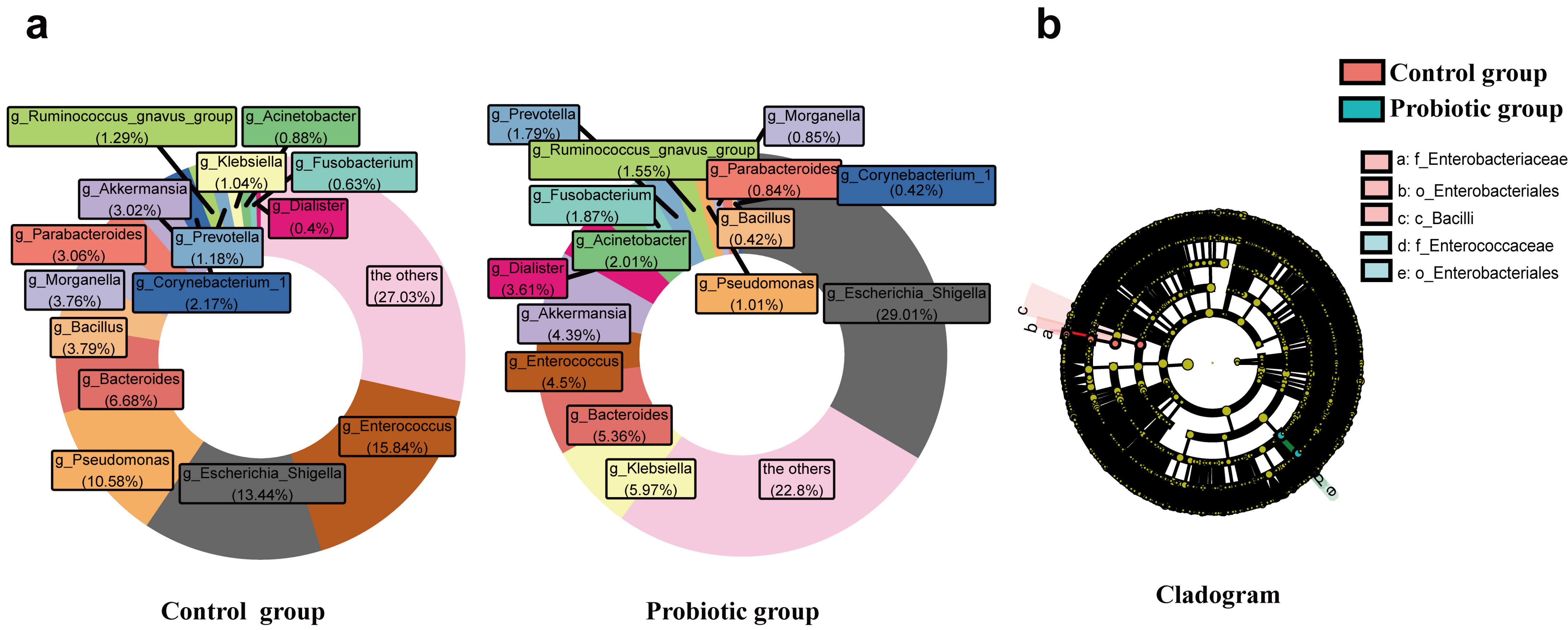


**Supplement Fig 6 Comparison of gut microbiota between Control group and Probiotic group. The diameter of each cicle's diameter is proportional to the taxon's abundance.**

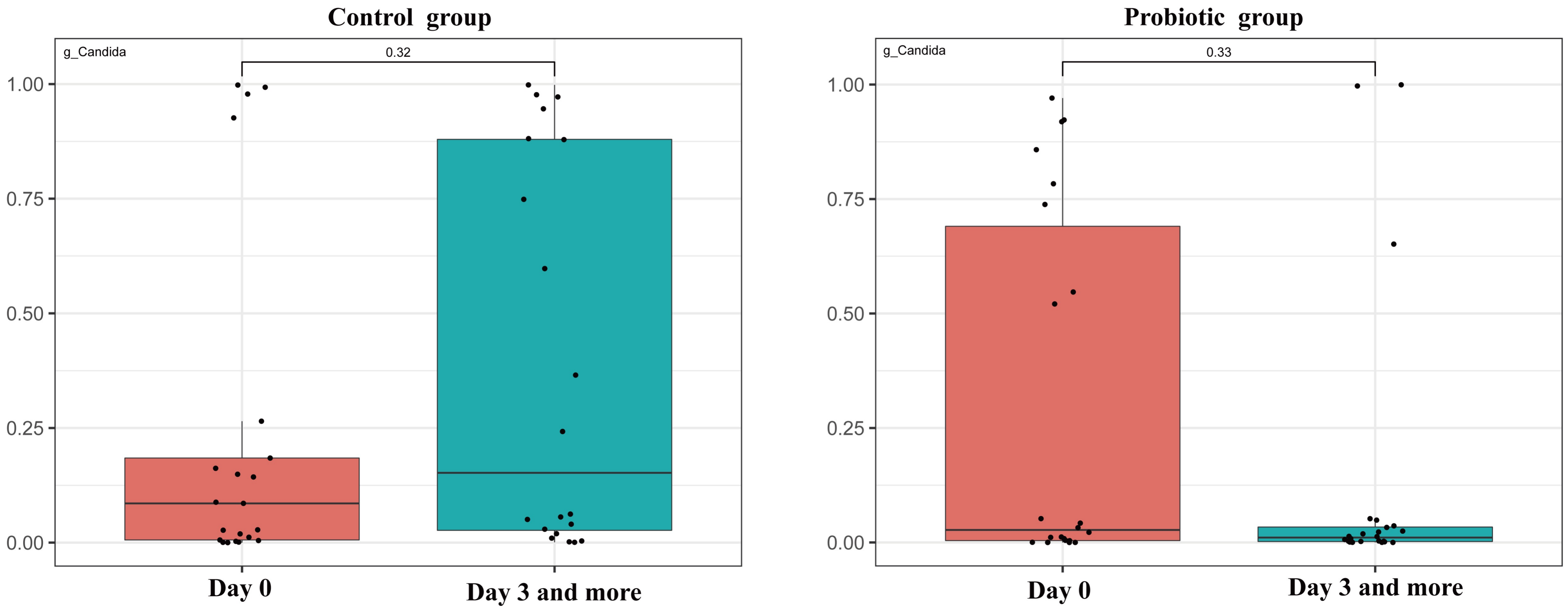


**Supplement Fig 7 Comparison of respiratory Candida proliferation beyond 48 hours after ICU admission between Control group and Probiotic group.**

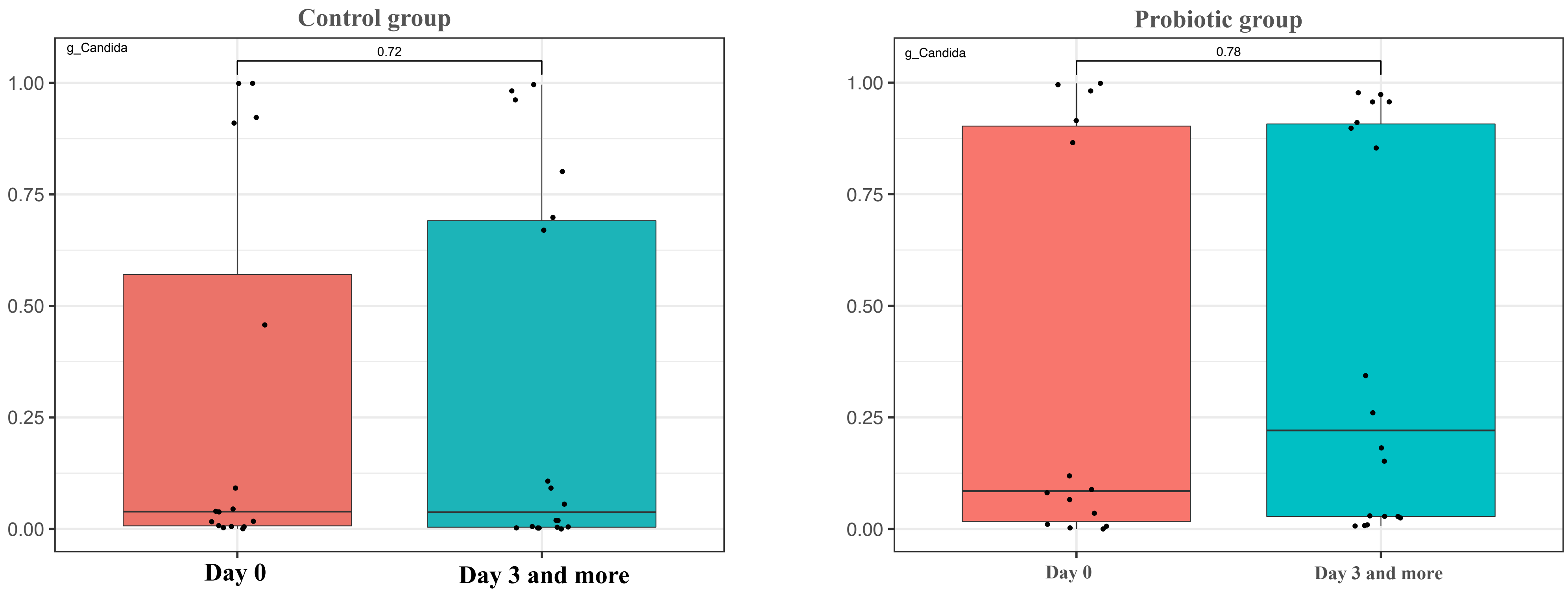


**Supplement Fig 8 The proliferation of candida in gut from administration into ICU and more than 48 hours between Control group and Probiotic group.**

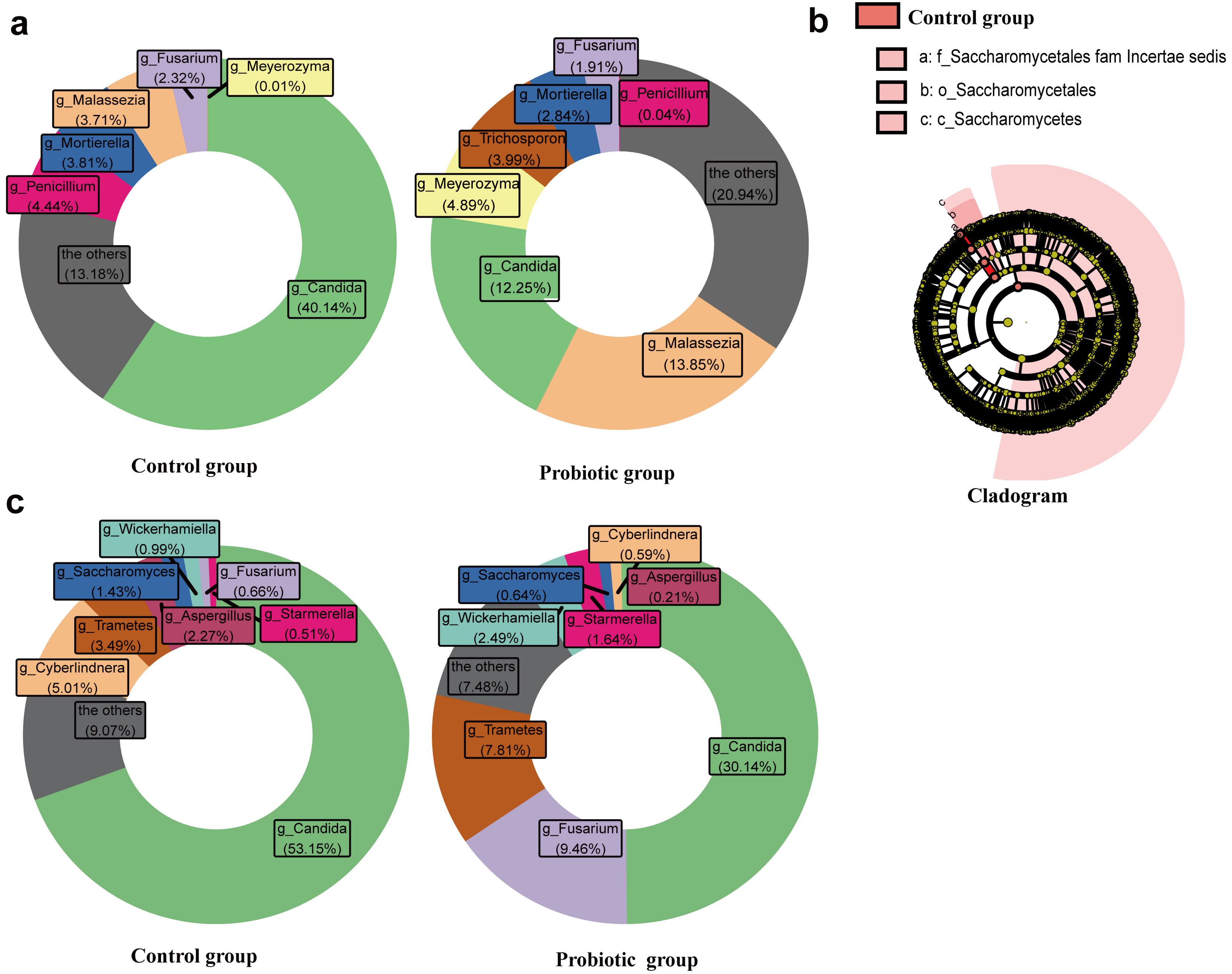


**Supplement Fig 9 Comparison of respiratory tract and gut mycobiota between Control group and Probiotic group. a.c The diameter of each cicle's diameter is proportional to the taxon's abundance.**
